# *In vitro* evaluation of bio-fortification effects on the nutritional quality, toxicological safety, and antioxidant of cassava (*Manihot esculenta*) flour, and environmental safety of processing water, using natural additives

**DOI:** 10.3389/fnut.2025.1731737

**Published:** 2026-02-03

**Authors:** Tasahil S. Albishi, D. Adaigho, Nashi K. Alqahtani, Rokayya Sami, Hilary Uguru, Mahmoud Helal, Shatha H. Alaoufi, Mohammed Saeed Alkaltham, D. Osiga-Aibangbee, G. I. Okolotu, E. P. Emumejaye, Sameer H. Qari

**Affiliations:** 1 Biology Department, College of Science, Umm Al-Qura University, Makkah, Saudi Arabia; 2 Department of Agricultural Economics, Southern Delta University, Ozoro, Nigeria; 3 Department of Food and Nutrition Sciences, College of Agricultural and Food Sciences, King Faisal University, Al-Ahsa, Saudi Arabia; 4 Department of Food Science and Nutrition, College of Sciences, Taif University, Taif, Saudi Arabia; 5 Department of Agricultural Engineering, Southern Delta University, Ozoro, Nigeria; 6 Department of Mechanical Engineering, Faculty of Engineering, Taif University, Taif, Saudi Arabia; 7 Department of Early Childhood, College of Education, Qassim University, Buraydah, Saudi Arabia; 8 Department of Food Science and Nutrition, Faculty of Food and Agricultural Sciences, King Saud University, Riyadh, Saudi Arabia; 9 Department of Physics, Delta State University, Abraka, Nigeria; 10 Department of Biology, Al-Jumum University College, Umm Al-Qura University, Makkah, Saudi Arabia

**Keywords:** antioxidant activity, bio-processing, heavy metals and cyanide toxicity, malnutrition, microbial action, public health

## Abstract

The impact of bio-fortification on the nutritional quality, anti-nutritional factors, glycemic indices, and toxicological effects of cassava (*Manihot esculenta*) flour (CF), was investigated in vitro in this scientific research. The cassava roots were administered with, eleven distinct treatment strategies (F1 to F11), formulated from *Moringa oleífera* leaves, *Syzygium aromaticum* seeds, and *Curcuma longa* rhizomes derivatives. Additionally, two control units were used: Control A, which consisted of unsoaked cassava roots; and Control B (the vehicle control), which involved soaking the cassava roots in untreated tap water. All the laboratory tests were conducted in accordance with standard procedures. The experimental outcomes indicated that, the treatments significantly impacted the physicochemical and biological properties of the CF, and the soaking water (p ≤ 0.05). Especially, the treatments contribute intensely to the CF antioxidant activity, and health safety of the fortified flour. It was observed that, the hybridized treatments resulted in a 69.76% reduction in the HCN levels, and a 51.28% reduction in the GI levels in the CF, respectively. Also, the treatments initiated nutrients infusion into the enriched CF, as the carotenoid level increased from 0.47 to 1.80 mg/100 g; protein content increased from 1.34 to 1.99%; carbohydrate content increased from 74.33 to 84.33 g/100 g DW; and B vitamin content increased from 0.37 to 0.87 mg/100 g DW. Though, the Control A flour contained appreciable essential nutrients contents, its high glycemic index potential (78), and hydrogen cyanide level (0.43 mg/100g DW), increases its health risk. Notably, the treatment resulted in 31.11% reduction in the total bacterial counts, of the soaking water, as well as a biodegradation of the toxic metals (Cd, Cr, As, and Pb) in the enriched flour. Particularly, the methodological approach adopted in this research will facilitate high eco-friendly cassava flour production, as well as the utilization of cheap and readily available bio-additives.

## Introduction

Malnutrition, which results from unhealthy diets and poor nutrition, is affecting billions of people globally, with children being much more vulnerable to the condition. Malnutrition conditions resulting from diet-related noncommunicable diseases - such as neurological disorders, cancer, stroke, hypertension, heart disease, diabetes, kidney dysfunction, and liver failure, are among the major killers of human beings (1–3). Salim et al. (4) stated that diets containing high amounts of anti-nutrients including phytates, oxalates, and tannins, can increase the occurrence of anemia, stunted growth, and bone diseases, as these nutrient antagonists impede the bioavailability of basic minerals in the body. Nutrient bioavailability significantly increases after bio-fortification, and the agricultural produce undergoes substantial biochemical modifications, resulting in increased concentrations of bioactive molecules in the diet (5).

Cassava (*Manihot esculenta*) is the leading root crop with high yield globally, and its products are staple foods for about 30% of the world population; hence, it plays a major part through the actualization of global food sufficiency (6, 7). Some staple food products obtained from cassava root include flour, gari (also spelled as garri), cassava chips, fufu, cassava rice, tapioca pearls, and starch (8). Cassava flour (CF) is widely utilized as partial replacement for wheat flour, in the bakery sector, due to its unique physicomechanical and biochemical properties. It is gluten-free, flavorless, and rich in polysaccharides; hence, it is blended with wheat flour and additional excipients to achieve high-quality bakery foodstuffs (9–11). Additionally, CF is a key component of infant food, instant foods, pastas, and sauces. According to Garavito et al. (12), cassava starch is a cost-effective production component in the food industry, mainly in bakery operations, primarily due to its affordability and availability. Borku et al. (13) reported that cassava flour has large amount of resistant starch, which helps improve digestive tract health, probiotic efficacy, and blood sugar monitoring. The prevention and management of Celiac disease, is a major reason that is contributing to cassava products’ popularity in the food industry. This is because this illness can be effectively managed with higher-fiber, gluten-free diets (9).

Although there are many benefits linked to cassava, cassava root is still considered to have a nutrient trade-off crisis, as it has high energy and carbohydrate content, but lacks proteins, vitamins and minerals (13–15). This nutrient trade-off is a principal challenge in the nutritional sector, as excessive consumption of un-fortified cassava products can result in malnutrition. Fortification of cassava flour with additives is one of the major steps, in improving its nutritional status. This helps in tackling the hidden hunger problem associated with excessive cassava intake (11, 14, 16). There are three major complementary approaches to enhancing the bioengineering and dietary attributes of cassava roots: breeding new crop varieties (genetic improvement), agronomic biofortification, and processing fortification. Enhancing cassava flour’s antioxidant activity is pivotal, in improving its nutritional and functional status, by preventing grave health issues such as coronary artery disease, diabetes, and degenerative brain diseases (5). These treatments (fortification) not only improve the nutritional and medical attributes of the cassava roots, but also degrade some toxic compounds linked to the cassava roots (2, 11, 17–19, 20). According to Nebiyu and Getachew (20), soaking or drying of cassava roots help reduces their cyanogenic glucosides content, prompting a decline in health risks associated with hydrogen cyanide (HCN) poisoning.

While benefits are evident pertaining to the utilization of CF in the food industry, it has some vital limitations, such as high glycemic index (GI) and hydrogen cyanide (HCN) values, which constitute substantial health threats to the consumers (5, 21, 22). People living with diabetes and metabolic disorders are allergic to diets containing high glycemic index values; as such diets worsen their condition by causing rapid spikes in blood glycemia (23, 24). Hydrogen cyanide toxicity includes failure of vital organs, loss of consciousness, thyroid dysfunction, neurological disorders, and developmental issues. These limitations can be effectively handled through adequate cassava roots processing operations such as fermentation, boiling, and drying. Nilusha et al. (8) recorded high HCN levels in various cassava flours, and recommended adequate processing operations for cassava roots to ensure public health and food safety.

Heavy metal toxicity is another major challenge in the food industry. Prolonged exposure to toxic metals such as lead (Pb), Thallium (Tl), cadmium (Cd), mercury (Hg), Nickel (Ni), chromium (Cr), and arsenic (As) poses substantial health hazards, particularly in infants and vulnerable adults (25–27). Over the decade, considerable emphasis has been placed on the bio-processing of cassava roots, to increase their nutrient bioavailability, medicinal quality, and degradation of HCN (5, 8, 21). However, there is no unified comprehensive research report that, simultaneously evaluates the bio-fortification of cassava flour (CF), through hybridization of three different plant extracts, coupled with monitoring the ecological friendliness of the processing water. This single experimental structure will assess the potential of multi bio additives hybridization, to degrade anti-nutrients and GI level in the enriched CF, improve essential nutrients and antioxidants in the processed CF, and promote biodegradation of toxic substances in the cassava roots processing water. Therefore, the major goals of this research, are to investigate the effects of hybridized moringa leaves, clove seeds, and turmeric rhizome derivatives on the following factors: (i) antioxidant (including nutrients) augmentation of the enriched CF; (ii) degradation of anti-nutrients, glycemic index, and toxic compounds in the CF; and (iii) enhancement of the eco-friendliness of the cassava processing operation, specifically the soaking water. Also, advanced statistical tools such as Principal Component Analysis, will be used to evaluate the impact of the treatments on food and public safety.

## Materials and methods

### Cassava roots

The mature cassava (cultivar TME 419) roots, grown under similar environmental conditions and cultural practices, harvested from the research farm of the Department of Agricultural Engineering, Delta State University of Science and Technology (DSUST), Nigeria, were used for this study. The cassava was planted in April 2024 and harvested in April 2025.

### Plant extracts production

The moringa leaves extract, clove seed extract, and turmeric rhizomes extract, were obtained from the bioprocessing laboratory, of the Department of Agricultural Engineering, DSUST. The extracts were prepared using ethanol, by following standard procedure. The plant materials were air-dried (24–30 °C) for 10 days, sorted to remove foreign bodies, and pulverized using a laboratory milling machine. Each plant material particulate was mixed with food-grade ethanol at a ratio of 1:10 (w/v). The container was hermetically sealed and left to stand for 5 days at room temperature (23 – 34 °C). The mixture was stirred gently for every 12 h, to enhance the effectiveness of extraction procedure. Subsequently, the filtrate was recovered from the mixture through filtration (using a Whatman No. 1 filter paper), and the extract was concentrated by drying it in a water bath at 40 °C (28, 29). This lower evaporating temperature was selected to avoid the thermal degradation, of the volatile phytochemicals (30). Basically, food grade ethanol (80% purity, CAS, 64–17-5, Molecular Weight of 46.069 g/mol) was selected over methanol (methyl alcohol), because of the toxicity associated with methanol, which is harmful to human beings (31).

### Chemicals and laboratory apparatus

All the chemicals used in this experimental investigation were of the analytical grade. DPPH (2,2-diphenyl-1-picrylhydrazyl) powder, ethanol, Folin–Ciocalteu reagent, nutrient agar (NA), and methanol were of the analytical grade and manufactured by Fisher Scientific, USA. Additionally, the UV–Vis spectrophotometer was produced by Thermo Scientific, Massachusetts, USA. The electronic scale (model: WT50001KF and accuracy of 0.1 g) was manufactured by WANT Balance Instrument Co., Ltd., China. The digital pH meter (model: GSI-011, resolution of 0.1 pH, and accuracy of +0.01pH) was produced by Globe Scientific Instruments India. The digital TDS meter (model: TDS 652, resolution of 0.1 ppm, and accuracy of ±0.5%) was manufactured by Electronics India company, India; while the BOD meter (Model: BOD-573, accuracy of BOD 5), was manufactured by Wincom Company Ltd., China. The HPLC system (model: Vanquish Flex UHPLC, max pressure of 100 MPa, flow rate range of 0.00 to 8.00 mL/min) was manufactured by Thermo Fisher Scientific, Massachusetts, USA.

### Experimental design

The investigational mix design ratios used in this research are presented in Table 1. The concentration of each plant additive was standardized comparative to the cassava product (roots or flour) used for the experiment. Specifically, 2% (w/w) indicates that 2% of the cassava roots/flour weight used for each experimental unit. The soaking solutions were prepared, by dissolving the appropriate amount of the treatment, in tap water at room temperature. Notably, the plant powders were not dissolved in water, but rather they were blended with the dried cassava flour. These lower CF blending ratios (1% plant powder and ≤12% plant extract), were adopted to prevent nutrients antagonism, safeguard heath safety, and promote collaboration with earlier documented research. Basically, the 11 treatments and the two controls will provide comprehensive data, which will aid ANOVA analysis, leading to better reliable results.

**Table 1:** The experimental mix design.

Treatment code	Constituents (w/w)
Control A	Un-soaked cassava roots (no treatment)
Control B (Carrier/vehicle control)	Cassava roots soaked in tap water
F1	1% CSP + 2% CSE
F2	1% CSP + 4% CSE
F3	1% TP + 2% TE
F4	1% TP + 4%TE
F5	1% MLP + 2% MLE
F6	1% MLP 4% MLE
F7	1% CSP + 2% CSE + 2%TE
F8	1% CSP + 2% CSE + 2% MLE
F9	1% TP + 2% TE + 2% MLE
F10	1% TP + 2% CSE + 2% TE + 2% MLE
F11	1% TP + 4% CSE + 4% TE + 4% MLE

CSP, clove seed powder; TP, turmeric rhizome powder; MLP, moringa leaves powder; CSE, clove seed extract; MLE, moringa leaves extract; TE, turmeric rhizomes extract.

Particularly, tap water was used in soaking the cassava roots this study instead of distill water, mainly due to these reasons: cost implications, ease of replication, and the soaking medium commonly used in previous studies. A large volume of water (about 200 L) is required for the soaking operation; therefore, the use of tap water will make the research cost-effective and viable, especially in low- and medium-income countries, where distilled water will not be easily accessible. Therefore, the utilization of the tap water, instead of distill water will enhance consistency and reproducibility of this research as well as factual household application. Additionally, since tap water does not undergo further treatments apart from filtration (unlike distilled water), it will contain some minerals, physicochemical, and biological attributes, that enhance microbial fermentation of the cassava roots. Similarly, borehole water has characteristics similar to tap water in this experimental setup, since the tap water used in this study is underground water (borehole water), which just undergoes filtration as the only treatment.

Moringa leaves were chosen due to their potent nutritional value (Flavonoids, vitamins proteins and minerals), as their essential compounds will enhance the nutritional quality, of the processed cassava flour (32). Clove seeds (*Syzygium aromaticum*) and turmeric rhizome (*Curcuma longa*) were chosen due to their high antioxidant activities, as these materials are rich in curcumin, flavonoids, phenolic acids, eugenol, gallic acid, and tannins (33, 34). This will help degrade toxic compounds (such as heavy metals and HCH), potentially reduce the GI level, and enhance the nutrient contents of the cassava flour. According to these scholars (35, 36), clove seeds, turmeric, and Moringa leaves contain large amounts of phytochemicals such as eugenol, curcumin, and moringinine, which are highly effective in inhibiting microbial growth. Apart from their nutritional and antioxidant values, these plant materials are easily accessible across the world, particularly in cassava-producing countries, where they are used for pharmaceutical and dietary applications. This eliminates the fear of food poisoning at lower concentrations, as utilized in this experimental mix design ratio in the practical workflow.

### Cassava flour preparation

The cassava roots were harvested at peak maturity (12 months after planting), peeled and washed physically with tap water. Then, the washed roots were inspected manually, to discard all rotten or pest-infested roots, as these damaged roots can interfere with the experiment’s results. Approximately 10 kg of the cleaned cassava roots were cut into medium pieces and completely immersed in 10 L of water. The soaking duration was 48 h at room temperature (24^o^ − 32 °C), without water replacement; and the water was gently agitated every 6 h, to prevent buildup of anaerobic pockets, improve uniform chemical reactions throughout the water. After soaking, the roots were sun-dried (32 ± 4 °C) for 24 h, and then oven dry (65 ± 1 °C) until the moisture content declined to approximately 8%. These drying parameters were chosen to preserve the nutritional values of the cassava roots, ensure the microbial safety of the cassava flour, improve grinding efficiency, and reduce power consumption. The dried roots were ground with a burr mill and screened with a 150 μm sieve to obtain the cassava flour. Then, the flour was blended with the right amount of plant powder, and was packaged in sealed, air-tight containers, since CF has a hygroscopic nature.

### Laboratory analysis

#### Water physicochemical and microbial analysis

The APHA (37) recommended procedures, were used to measure the Water physicochemical parameters. The water pH and total dissolved solids (TDS) levels were measured, by using the digital pH meter and digital TDS meter, respectively; and the Biochemical oxygen demand (BOD) level of the water samples was measured with a digital BOD meter. Basically, the total bacterial count (TBC) of the water was measured, by using the plate count method, with nutrient agar and an incubation temperature of 32 °C for 2 days.

Additionally, the Atomic Absorption Spectrophotometry (AAS), was used to measure the concentration of Lead (Pb), Cadmium (Cd) Arsenic (As) Chromium (Cr) in the water samples. The water was collected with sterilized bottles, Acidified using a few drops of nitric acid (HNO_3_), and sieved using the 0.45 μm membrane. The following analytical wavelengths - 217.0 nm, 228.8 nm, 193.7 nm, and 357.9 nm, were used to measure the absorbance of Pb, Cd, As, and Cr, respectively.

### Anti-nutrients determinations

#### Phytate content

The CF samples phytate content was determined by the Wade reagent colorimetric technique, by using a spectrophotometer. 5 g of the CF was mixed with 20 mL of HCl (0.2 N), which was allowed to digest for 3 h at 27 ± 3 °C, before it underwent centrifugation at 3500 g for 15 min, and the clarified liquid was sieved by utilizing a Whatman No. 1 filter paper. Additionally, the standard solutions (sodium phytate) were prepared using 0.2 N HCl. Then 3 mL of both the specimen and standard, were mixed thoroughly with 1 mL of the Wade reagent, and left for 10 min at 27 ± 3 °C, before its absorbance was measured with a spectrophotometer at 500 nm. The CF phytate content was computed by using Equation 1.


(1) $$\text{Phytate content}=\frac{c\times v_{e}\times \mathrm{DF}}{1000\times m}$$

C - phytate concentration obtained using the standard curve, Ve – sample volume, DF - dilution factor, m - sample mass.

#### Oxalate concentration

The CF samples oxalate content was determined through the titrimetric technique. Exactly 2.0 g of the CF was added to 100 mL of 1 M HCl, tremble for 90 min at 27 ± 3 °C, and sieved using a Grade 1 filter paper, to recover the digested extract. 100 mL of the extract was mixed with 10 mL of CaCl_2_ solution (5% w/v), stirred thoroughly, and the calcium oxalate precipitate was recovered, washed with distilled water, and treated with 25 mL of diluted H_2_SO_4_ (1 M). This product was heated at 80 °C in a water bath, until a transparent solution was attained, and was rediluted with distilled water to 100 mL. 25 mL of the aliquot was titrated against standardized 0.05 M KMnO_4_ solution, and the CF oxalate value was calculated, which was expressed as mg/100 g dry weight (DW) basis.

#### Tannin concentration

The CF tannin concentration was determined using the Folin–Denis spectrophotometric approach according to AOAC (38) guidelines. 1 g of the CF was digested with 20 mL of 70% (v/v) aqueous acetone, at approximately 25 °C for one hour. The mixture was then spun at 3000 × g for 20 min to produce the extract. Then, 2 mL of the extract was combined with 10 mL deionized water, 1 mL Folin–Denis reagent, and 2 mL saturated Na_2_CO_3_, after which incubation was performed at 25 °C for 40 min. The solution’s absorbance was recorded with a UV–Visible spectrophotometer at 760 nm, and the tannin concentration of the CF was expressed as mg TAE/100 g dry weight (DW) basis (39).

#### Hydrogen cyanide concentration

The HCN level in the flour samples was determined in accordance with the AOAC (38) Official approach, using the distillation and titration approach, as described by Nilusha et al. (8). The hydrogen cyanide concentration in each titrated sample was then computed using Equation 2 (25).


(2) $$\mathrm{HCN}=\frac{(\text{titre}-\text{Blank})\times 1.08\times 0.02}{\text{sample weight}}$$

### Nutritional quality determination

#### Carotenoids concentration

The CF total carotenoid content was measured via a spectrophotometric approach, measuring absorbance at 450 nm using a UV–Vis spectrophotometer, with petroleum ether serving as baseline. The CF extract was prepared using acetone and petroleum ether (mixed at a ratio of 1:1), and the mixture was homogenized thoroughly for 20 min. Also, concentrated NaCl solution was used to inhibit emulsion formation during the testing period (16).

#### The total B vitamins concentration

The total B vitamins in the CF were measured by the High-Performance Liquid Chromatography (HPLC) method, in accordance with AOAC (38) guidelines. The operating parameters of the HPLC system were: a flow rate of 1 mL/min, an injection volume of 10 μL, a reverse-phase C18 column, a column temperature of 26 °C, a mobile phase of methanol: phosphate buffer, a run time of 25 min, and a detection wavelength of 240–295 nm. Then, the quantification of B vitamins (B1, B2, B3, B6, and B9) was done by using standard calibration curves. The Correlation coefficient (R^2^) value was 0.998, the limit of detection (LOD) was 0.02 μg/mL, limit of quantification (LOQ) was of 0.10 μg/mL, recovery rate values varied from 94 to 105%, and Relative Standard Deviation (RSD) values were below 4%.

Remarkably, methanol (99.8% purity, molecular weight 32.04 g/mol, CAS, 67–56-1) was used as a solvent for all the HPLC analyses, mainly due to its lower flow resistance compared to ethanol. Also, methanol is the widely recommended AOAC solvent for HPLC protocols, as it provides better peak resolution and has a lower UV absorbance, when compared to ethanol (40).

#### Vitamin C (ascorbic acid) concentration

The CF vitamin C concentration was determined through a titrimetric approach using 2,6-dichlorophenolindophenol as an indicator. Ten grams of the CF was incorporated into 100 mL of metaphosphoric acid (MPA), homogenized for 20 min, centrifuged at 3,000 g for 20 min, and filtered to recover the supernatant. 10 mL of the digested extract was titrated against MPA, in the presence of the indicator until an endpoint (persisted faint pink coloration) was achieved. Then, the vitamin C level in the solution was calculated, and results were expressed in mg/100 g.

#### Total vitamin E concentration

The total vitamin E content of the CF was determined using the HPLC approach, equipped with C18, 250 × 4.6 mm column. The HPLC operating parameters were: 20 μL injection volume; 1.0 mL/min flow rate; mobile phase of methanol:water; 30 °C operating temperature; detection wavelength of 292 nm; and run time of 25 min. Typically, the HPLC operation has these specifications: R^2^ value of 0.998, LOD and LOQ values of 0.010 and 0.060 μg/mL, precision value lower than 4%, and the CRM recovery rate ranges from 94 to 105%. Also, the standard concentration varied from 0.005–10.000 μg/mL.

#### Carbohydrates concentration

The carbohydrates concentration of the cassava flour was evaluated, by utilizing the difference technique, as explained by Nilusha et al. (8). The carbohydrate concentration was calculated via Equation 3.


(3) $$\begin{array}{l} \text{Carbohydrate}=\\ \;\;100–(\%\mathrm{MC}+\%\mathrm{PC}+\%\mathrm{FC}+\%\mathrm{AC}+\%\text{fiber content}) \end{array}$$

Where: MC – moisture level, PC – crude protein, fat content, AC - ash content.

### Fat determination

The CF fat content was determined using Soxhlet extraction, following AOAC (38) guidelines.

### Protein determination

The Kjeldahl nitrogen method was utilized to assess the protein concentration, following the AOAC (38) guidelines as explained by Nilusha et al. (8). 1.0 g of the CF was added to 20 mL of H_e_SO_4_ in a Kjeldahl flask, and digested in the presence of selenium as a catalyst, until a clear solution was attained. The ammonia content liberated from the solution, by the addition of NaOH was titrated against HCl, and the protein level in each specimen was computed by using Equation 4.


(4) Protein(%)=N×6.25

Where N - calculated nitrogen value.

### Antioxidants determinations

#### Total phenolic content concentration

The flour samples’ TPC levels were determined by using the Folin–Ciocalteu assay approach, as recommended by AOAC (38), and explained by Nilusha et al. (8). 1.0 g of the CF was integrated to 10 mL of methanol, vortexed for 1 min, and subjected to centrifugation for 10 min at 3500 × g. The solution was sieved through a 0.45 μm membrane filter, and reconstituted with distilled water 10 mL. Thereafter, 1.0 mL of the diluted assay was mixed with 2.0 mL of Folin–Ciocalteu reagent and left undisturbed for 5 min, before 2.0 mL of Na₂CO₃ (7.5%) solution was added it, and incubated at 25 °C for 70 min. The final product absorbance was determined at 750 nm, by using a UV–Vis spectrophotometer. The PC of each CF sample was expressed as mg gallic acid equivalents (GAE) per 100 g of dry weight (mg GAE/100 g DW) (5).

#### DPPH radical scavenging activity concentration

The cassava flour DRSA level was determined in agreement with approved guidelines as described by Nilusha et al. (8). The CF extract was produced by adding 1 g of the CF to 20 mL methanol, and subjecting the mixture to centrifugation at a speed of 4,000 × g for 15 min. Then, 0.2 mL of the extract was incorporated into 1 mL of DPPH solution, incubated at 27 °C for 30 min, and the absorbance value was assessed with a UV–Vis spectrophotometer at 517 nm. The DRSA was computed through Equation 5.


(5) $$\%\text{inhibition}=\frac{A_{\text{control}}-A_{\text{sample}}}{\text{Acontrol}}\times 100$$

#### Glycemic index concentration

The *in vitro* (laboratory digestion) method was used to predict the GI level in the flour samples. 100 mg of the boiled flour was poured into the digestion tube, and pancreatin and amyloglucosidase were added at 34 °C. Glucose levels in the aliquots were tested at regular intervals and were converted to starch hydrolyzed using a factor of 0.9. To determine the predicted GI, the amount of starch hydrolyzed was plotted against time, and the GI was calculated using Equations 6 and 7 (41).


(6) H1=AUCRefAUCsample×100

(7) GI=39.71+0.549×HI

Where: HI - Hydrolysis Index, AUC - area under the curve.

### Toxicological determination

#### Heavy metals concentration

The Pb, Cd, Cr and As concentrations in the CF were determined in harmony with ASTM-approved procedures. Five grams of the CF were added to concentrated HNO_3_ and HCl acids (mixed at a ratio of 4:1) in a digestion flask, and heated with a hot plate until digestion was completed. Then, the Pb, Cd, Cr, and As concentrations in the digested solution was measured, using an atomic absorption spectrophotometer (AAS) at each metal’s specified wavelength.

### Statistical analysis

The results gotten from this scientific investigation were evaluated through analysis of variance (ANOVA), to determine if the treatments have significant influence on the cassava flour. Also, the mean values obtained were separated, by employing the Duncan’s Multiple Range Test (DMRT) tool, at the 5% significance level. Each test was performed three times, and the averages and standard deviations were recorded. These results were presented in tables and figures. Additionally, the Principal Component Analysis (PCA) statistical tool, developed with Python software as used, in results visualization, along with further interpretation of the results.

## Results and discussion

### Soaking water quality analysis

The results of the tap water, and the soaking water recovered after the 48-h soaking are presented in Tables 2, 3. As seen in Tables 2, 3, the microbial load, physicochemical characteristics, and heavy metals (HMs) content of the water varied widely, and these factors were substantially influenced (*p* ≤ 0.05), by the treatment options (F1–F11). This supports previous reports of Olaniyan et al. (42), which indicated that the leachate from cassava root, substantially affected the microbial load, and the physicochemical properties of water and the environment. The low bacterial counts in the tap water (2.21 log_10_ cfu/mL), was drastically increased to 2.59 log_10_ cfu/mL, after soaking the cassava roots in untreated water. This can be linked to organic effluent discharged from the roots into the water, which enhanced microbial activity, through the provision of essential nutrients and suitable environmental conditions (43). It was observed that the TBC recorded in the plant extracts treated water, decreased dramatically, as the treatment concentrations increases. This signifies the antimicrobial effect of the extracts used in the bio-fortification process. Eugenol and curcumin have a strong ability to inhibit bacterial growth, by increasing cell wall absorptivity level, which disrupts nutrient utilization and ultimately leads to cell lysis (44, 45). Also, the phytochemicals such as moringinine and phenolic acids, present in the treatments are very effective, in inhibiting peptidoglycan production. This leads to the formation of bacterial cells with very fragile and porous cell membranes, hence exposing the cells to osmotic shock (46).

**Table 2:** The microbial and physiochemical properties of the cassava roots soaking water.

Sample code	TBC (log_10_ cfu/mL)	pH	BOD (mg/L)	TDS (mg/L)
Tap water	2.21^a^ ± 0.06	7.37^g^ ± 0.06	23.33^a^ ± 2.52	219.00^a^ ± 10.15
Control B	3.76^j^ ± 0.03	7.13^fg^ ± 0.06	62.00^b^ ± 2.65	325.33^abc^ ± 11.93
F1	3.10^f^ ± 0.06	6.33^cd^ ± 0.15	142.00^ef^ ± 4.36	439.00^cde^ ± 24.43
F2	2.99^e^ ± 0.02	6.13^c^ ± 0.06	162.67^g^ ± 4.16	521.00^de^ ± 37.72
F3	3.26^h^ ± 0.06	6.63^e^ ± 0.15	135.67^e^ ± 3.06	396.33^bcd^ ± 11.93
F4	3.11^f^ ± 0.04	6.43^de^ ± 0.12	169.67^gh^ ± 7.23	448.33^cde^ ± 14.57
F5	3.35^i^ ± 0.06	6.90^f^ ± 0.10	103.33^c^ ± 7.77	349.67^bc^ ± 22.37
F6	3.24^g^ ± 0.02	7.00^f^ ± 0.17	125.33^d^ ± 3.51	284.33^ab^ ± 207.36
F7	2.83^c^ ± 0.05	6.10^bc^ ± 0.10	173.33^h^ ± 3.79	533.00^e^ ± 9.64
F8	2.92^d^ ± 0.03	6.47^de^ ± 0.15	164.33^g^ ± 5.69	492.33^de^ ± 15.63
F9	2.97^e^ ± 0.06	6.47^de^ ± 0.21	193.00^i^ ± 5.57	454.33^cde^ ± 10.69
F10	2.66^b^ ± 0.03	5.87^b^ ± 0.21	148.00^f^ ± 2.65	674.33^f^ ± 18.45
F11	2.59^b^ ± 0.07	5.33^a^ ± 0.15	122.00^d^ ± 3.61	744.33^g^ ± 21.76

Mean ± standard deviation; *n* = 3; in the sample parameter, rows have similar alphabet indicates that the means are not significantly different.

**Table 3:** The water heavy metals concentration of cassava roots soaking water (ug/kg).

Sample code	Cd	As	Pb	Cr
Tap water	0.00a ± 0.00	0.00^a^ ± 0.00	0.00^a^ ± 0.00	1.67^a^ ± 1.53
Control B	5.67^b^ ± 1.53	0.00^a^ ± 0.00	4.67^b^ ± 2.52	5.67^ab^ ± 2.08
F1	15.67^cd^ ± 2.08	0.40^bc^ ± 0.10	9.33^cde^ ± 3.06	9.67^bc^ ± 3.51
F2	21.33^e^ ± 2.52	0.67^cd^ ± 0.21	15.00^fg^ ± 3.61	13.67^cde^ ± 2.08
F3	15.00^cd^ ± 2.00	0.23^ab^ ± 0.21	7.00^bc^ ± 2.00	11.33^cd^ ± 1.53
F4	14.67^cd^ ± 3.06	0.60^cd^ ± 0.17	11.67^def^ ± 1.15	11.67^cde^ ± 3.21
F5	15.67^cd^ ± 1.53	0.07^a^ ± 0.00	7.00^bc^ ± 2.00	6.00^b^ ± 2.65
F6	12.67^c^ ± 2.08	0.23^ab^ ± 0.12	8.33^bcd^ ± 2.08	9.67^bc^ ± 2.08
F7	22.00^e^ ± 1.00	0.93^de^ ± 0.32	18.33^gh^ ± 3.06	14.67^de^ ± 2.08
F8	17.67^d^ ± 1.53	0.77^de^ ± 0.21	15.67^fg^ ± 1.53	13.33^cde^ ± 1.53
F9	15.67^cd^ ± 2.52	0.77^de^ ± 0.25	13.00^ef^ ± 3.00	11.67^cde^ ± 2.08
F10	27.67^g^ ± 1.53	1.10^ef^ ± 0.26	20.00^g^ ± 2.00	16.00^ef^ ± 2.65
F11	33.33^h^ ± 2.52	1.27^f^ ± 0.21	25.00^h^ ± 2.65	19.33^f^ ± 3.51

Mean ± standard deviation; *n* = 3; if rows have a common alphabet in the same column, then the means did not differ significantly (*p* ≤ 0.05).

Furthermore, it was noted that the water pH becomes more acidic, in the presence of the treatments, and the water acidity was approximately directly proportional to the treatment’s concentration. This scenario can be attributed to the organic acids and other phenolics, discharged by the treatments and cassava roots into the water. The elevated water acidity recorded in treatments F10 and F11, will have antimicrobial effects, because a lower pH environment suppresses most microbial functionality (47). High water acidity helps to increase metal solubility and mobilization (48), and this can be connected to the higher concentrations of HMs and TDS, recorded in the water from the treatment units, particularly in treatments F8 to F11. The non-linear increase in the water BOD level can be linked to the antimicrobial effects, initiated by existence of pharmacologically active constituents in the extracts, leading to a reduction in microbial utilization of organic materials (49). Remarkably, the concentrations of Cd, Pb, Cr, and As in the water, increased as the treatment concentration increases, with the optimal levels recorded in the F10 and F11 samples (Table 3). This major drawback (from the environmental toxicological perspective), can be attributed to the favorable conditions provided by the extracts, in dissolving and leaching metallic compounds from the cassava roots; however, this situation may have a positive impact on the cassava roots, as some of the toxic metals will be degraded, resulting in safer cassava-based diets. This study’s findings have underscored the impact of plant extracts on water used in agricultural processes, particularly cassava root fermentation, and underscore the paramount need for proper remediation before discarding into the environment to protect public health.

### Anti-nutrients factors

The concentrations of the phytates, oxalates, tannins, and HCN in the CF samples, which were subjected to different treatments (F1–F11), along with the two control samples, Control A and Control B, are presented in Table 4. It was noted that the treatments have significant effects on the anti-nutritional parameters (*p* ≤ 0.05). Conspicuously, this study’s results indicate that, the plain water soaking process minimized anti-nutrient contents in the cassava flour. This aligned with these authors (50, 51) reports, which documented that soaking is a food processing method that helps to reduce the phytates, oxalates, and tannins in plant materials by leaching these compounds into the standing water. The HCN levels recorded in this study were less than those reported by Nebiyu and Getachew (20) for three cassava varieties. The reduction in the concentration of anti-nutrients observed in the Control B sample can be attributed to the activities of naturally occurring fermenting microbes, such as *Lactobacillus, Leuconostoc,* and *Saccharomyces* species, which are present in cassava roots. These microorganisms aid in the degradation of harmful compounds, plus enhance the biotransformation process (5, 17).

**Table 4:** The anti-nutrients content of the CF samples (mg/100 g DW).

Sample code	Phytates	Oxalates	Tannins	HCN
Control A	493.33^i^ ± 10.97	421.33^h^ ± 3.51	100.00^g^ ± 3.00	0.43^i^ ± 0.02
Control B	249.67^a^ ± 5.03	191.33^a^ ± 3.51	35.67^a^ ± 3.06	0.27^h^ ± 0.04
F1	271.67^bc^ ± 6.11	242.00^bc^ ± 3.00	58.33^c^ ± 1.53	0.22^f^ ± 0.03
F2	297.67^d^ ± 4.16	266.33^cd^ ± 5.51	64.67^d^ ± 3.51	0.21^e^ ± 0.02
F3	265.67^b^ ± 2.08	217.00^ab^ ± 3.61	47.67^b^ ± 1.15	0.24^f^ ± 0.02
F4	276.67^bc^ ± 4.51	226.33^b^ ± 5.03	53.67^c^ ± 3.06	0.21^e^ ± 0.03
F5	310.33^de^ ± 5.51	287.33^de^ ± 4.04	48.67^b^ ± 3.51	0.18^d^ ± 0.03
F6	346.00^g^ ± 21.79	269.00^cd^ ± 53.73	56.33^c^ ± 2.52	0.13^b^ ± 0.02
F7	282.33^c^ ± 2.52	313.33^ef^ ± 2.52	66.67^d^ ± 2.08	0.19^d^ ± 0.02
F8	326.33^f^ ± 4.51	327.33^f^ ± 4.73	77.33^e^ ± 2.52	0.16^c^ ± 0.03
F9	319.00^ef^ ± 2.65	324.33^f^ ± 4.51	74.67^de^ ± 2.52	0.16^c^ ± 0.03
F10	327.00^f^ ± 4.58	386.00^g^ ± 6.24	78.00^e^ ± 2.65	0.13^b^ ± 0.03
F11	363.00^h^ ± 4.58	405.00^gh^ ± 11.53	92.00^f^ ± 4.36	0.11^a^ ± 0.03

Mean ± standard deviation; rows with common letters - same column - indicate no significant difference in the means (*p* ≤ 0.05); *n* = 3; HCN, hydrogen cyanide; DW, dry weight basis.

Furthermore, the findings highlighted that the CF displayed a progressive range of phytates, oxalates, and tannins concentrations, with treatments F1 to F4 showing lower oxalate contents, while treatments F8 to F11 exhibited higher oxalate contents. F3 and F4 exhibited the lowest phytates, oxalates, and tannins concentrations, nearly matching the results obtained in the carrier control sample. Conversely, hybridized treatments F10 and F11 exhibited the maximum phytates, oxalates, and tannins concentrations. This is an indication that phytochemical enrichment plays an essential role in accumulation of the anti-nutrients in the cassava flour. The significant increase in the levels of CF phytates, oxalates, and tannins can be attributed to two major mechanisms: plant extracts leaching into the water, which leads to an increase of these compounds concentration in the soaking water, and the phytochemical infusion process, which helps to increase the anti-nutrient content in the root’s skin through diffusion (52). Additionally, incorporating 1.0% plant powder into the milled flour will help increase the concentrations of phytates, oxalates, and tannins in the CF. Clove, turmeric and moringa are rich in phytochemicals - such as tannins and other phenolic compounds, help increase the concentrations of phytates, oxalates, and tannins in the treated material, by infusing these compounds into the roots (50, 53–55). Also, the decline in the HCN concentration of the soaked-processed CF, may be linked to the hydrolysis of cyanogenic glucoside in the process, which is enhanced through enzymatic and phytochemical actions (20, 21). This reaction was facilitated by the plant extracts in the soaking water, as supported by the treatment results.

Most importantly, the experimental outcomes revealed that, the treatments were able to suppress the flour’s HCN levels within the recommended threshold of 10 mg/kg DW (1 mg/100 g DW) by FAO/WHO, basically for cassava and its derived products, which is considered safe for human consumption (56). This research HCN levels were significantly smaller, than the values documented by Anim et al. (25). The consumption of excessive phytates, oxalates, and tannins impairs the bioavailability of most essential nutrients by producing indigestible substrates that adhere to minerals and proteins in the diet; however, at lower concentrations, they exhibit beneficial antioxidant and antimicrobial properties (53, 55, 57). Phytates tend to inhibit iron absorption, oxalates have the potential to increase kidney stone risk, and tannins can inhibit protein digestibility (58). Hydrogen cyanide toxicity includes respiratory failure, coma, tropical ataxic neuropathy, Konzo, and histotoxic hypoxia (59).

### Nutritional quality

The results of the CF’s vitamins and proximate components are presented in Table 5. The data revealed that the soaking process, and the treatment interventions, has a significant influence on the nutritional quality of the cassava flour (*p* ≤ 0.05). Notably, the proximate components of the CF significantly increased with increasing treatment concentration across all the extracts used. As seen in Table 5, apart from the fat content, the nutrients levels varied widely in the CF samples, with the lowest values recorded in Control B, and the maximum values documented in the F11 CF specimen. The increment in the enriched CF nutritional quality can be associated, with the phytochemical contributions from the plant extracts. Phytochemicals such as eugenol, flavonoids, saponins, moringinine, and curcuminoids, enhance the nutritional value of the enriched CF through three major pathways. They improve the permeability of cassava root cells, thus encouraging the diffusion of essential nutrients from the soaking water into the root tissues, as the treated soaking water tends to contain higher nutrients content. Additionally, they facilitate the infusion of nutrients into the CF during blending, and also help retard nutrient degradation during the CF processing unit operations (5, 60–63).

**Table 5:** The nutritional quality content of the CF samples.

Sample code	Carotenoids (mg/100 g DW)	B vitamins (mg/100 g DW)	Vitamin C (mg/100 g DW)	Vitamin E (mg/100 g DW)	Carb (g/100 g DW)	Protein (%)	Fat (%)
Control A	1.00^d^ ± 0.14	0.63^f^ ± 0.04	1.67^cd^ ± 0.21	0.09^cde^ ± 0.02	88.00^h^ ± 1.00	1.58^h^ ± 0.04	0.55^g^ ± 0.02
Control B	0.38^a^ ± 0.03	0.21^a^ ± 0.04	0.70^a^ ± 0.10	0.04^a^ ± 0.01	74.33^a^ ± 1.53	1.05^a^ ± 0.04	0.54^fg^ ± 0.01
F1	0.47^a^ ± 0.06	0.37^c^ ± 0.03	1.43^bc^ ± 0.21	0.08^cd^ ± 0.01	78.33^cd^ ± 0.58	1.34^bc^ ± 0.04	0.52^ef^ ± 0.01
F2	0.61^b^ ± 0.05	0.45^d^ ± 0.02	2.03^def^ ± 0.35	0.10^de^ ± 0.01	80.33^de^ ± 1.15	1.43^ef^ ± 0.03	0.50^de^ ± 0.01
F3	0.82^c^ ± 0.03	0.28^b^ ± 0.03	1.10^b^ ± 0.20	0.06^ab^ ± 0.01	75.33^ab^ ± 1.15	1.29^b^ ± 0.03	0.53^fg^ ± 0.01
F4	0.93^cd^ ± 0.02	0.33^bc^ ± 0.03	1.77^cde^ ± 0.31	0.07^b^ ± 0.02	76.67^bc^ ± 1.53	1.35^cd^ ± 0.03	0.50^de^ ± 0.02
F5	1.18^e^ ± 0.03	0.43^d^ ± 0.02	2.47^gh^ ± 0.21	0.10^de^ ± 0.02	78.33^cd^ ± 1.53	1.40^de^ ± 0.03	0.53^fg^ ± 0.01
F6	1.39^f^ ± 0.05	0.52^e^ ± 0.03	2.70^hi^ ± 0.26	0.12^ef^ ± 0.02	80.33^de^ ± 0.58	1.48^fg^ ± 0.03	0.53^fg^ ± 0.01
F7	0.68^b^ ± 0.09	0.43^d^ ± 0.02	2.10^efg^ ± 0.20	0.14^f^ ± 0.02	78.67^cd^ ± 1.15	1.45ef ± 0.02	0.49^cd^ ± 0.01
F8	0.91^cd^ ± 0.02	0.56^e^ ± 0.05	3.07^i^ ± 0.21	0.14^f^ ± 0.01	82.00^ef^ ± 1.00	1.56^h^ ± 0.03	0.47^bc^ ± 0.02
F9	0.98^d^ ± 0.04	0.52^e^ ± 0.01	2.30^fgh^ ± 0.20	0.18^g^ ± 0.01	80.33^de^ ± 1.15	1.53^gh^ ± 0.03	0.49^cd^ ± 0.02
F10	1.52^g^ ± 0.04	0.77^g^ ± 0.06	3.10^i^ ± 0.26	0.21^h^ ± 0.02	83.33^fg^ ± 0.58	1.74^i^ ± 0.04	0.46^b^ ± 0.01
F11	1.80^h^ ± 0.06	0.87^h^ ± 0.04	3.63^j^ ± 0.15	0.24^i^ ± 0.02	84.33^g^ ± 1.15	1.99^j^ ± 0.05	0.43^a^ ± 0.02

Mean ± standard deviation; *n* = 3; Rows with common letters in the same column indicate no significant difference in the means (*p* ≤ 0.05). DW, dry weight basis.

This study’s findings corroborated reports published by previous scholars, which stated that soaking cassava roots and other plant materials, in plain water leads to a decline in their nutritional value, as a consequence of leaching and diffusion of nutrients into the immersion solution (64, 65, 103). The carotenoid levels documented in this *in vitro* investigation were greater than the results, published by Taleon et al. (64) and Odoemelam et al. (16) from similar processing methods. This study vitamin C and protein results corroborate the previous research findings of Lu et al. (9); while they were higher compared to the review results documented by Bayata et al. (66). Otondi et al. (2) reported that integrating 20 to 60% of chia seed (*Salvia hispanica* L.) flour, into cassava flour helps to increase the nutritional content of the enriched CF, and this is similar to the findings obtained in this research. Also, the protein, carbohydrates, and fat contents achieved in this research, were far less than the outcomes reported by Katunzi-Kilewela for cassava flour blended with 5–25% of chia seed flour. The protein and carbohydrate levels recorded in this current study, align with the findings of Widowati et al. (11), regarding CF produced from microorganisms-assisted fermented cassava roots. Similarly, Nilusha et al. (8) reported similar findings regarding protein and carbohydrate content, for CF processed through soaking treatment.

Also, during the soaking process, microbial actions will enhance the degradation of volatile bioactive compounds, macronutrients, and other nitrogenous compounds, leading to a reduction in the nutritional quality of the root (11, 67). Remarkably, the smaller fat contents achieved in the enriched CF, particularly F10 and F11 enriched flour samples, are advantageous, as lower fat levels facilitate prolonged shelf life, reduce rancidity, and improve the stability of flour-based diets, coupled with preventing the incidences of obesity and cardiovascular hazards. The results indicated that the processing enriched the CF carotenoid levels, thereby enhancing their potential to combat illnesses linked to vitamin A deficiency. Vitamins C and E are antioxidants that immensely help to neutralize free radicals in the body, enhance immune responses, and improve enzymatic performance, thus playing essential roles in preventing oxidative stress in the body (68, 69).

### Heavy metals content

**Figure 1:**
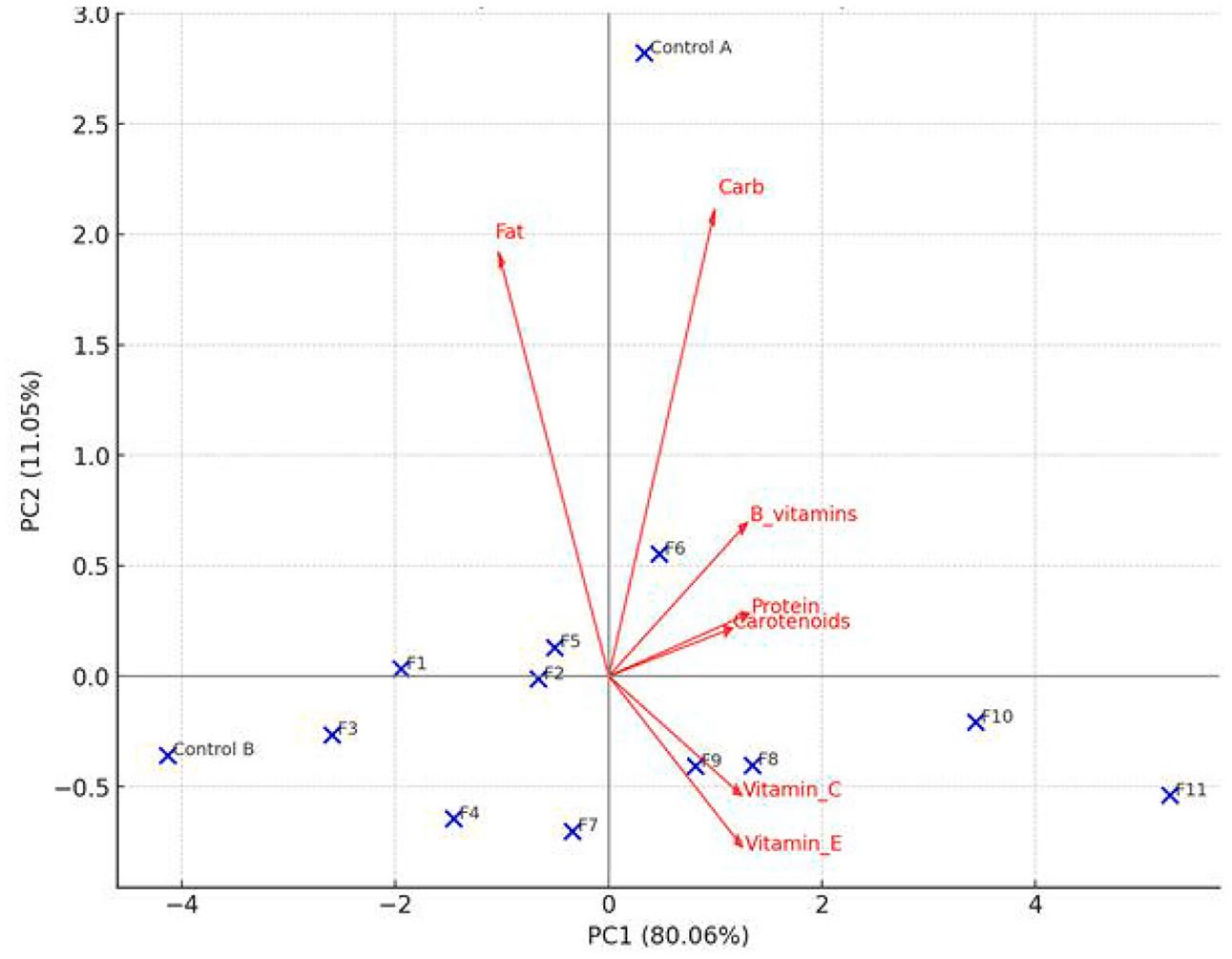
The PCA biplot of the macronutrient and micronutrient parameters of the cassava flour.

The results of the HMs concentration in the CF are presented in Table 6, and Figure 1. The results revealed that the treatments have a significant influence on the CF heavy metals content (*p* ≤ 0.05). Generally, Control A exhibited the highest heavy metals concentration, while F11 recorded the lowest HMs levels. This indicates that the bio-fortification treatments significantly curtailed all the levels of HMs. The results revealed that, the treatments were more effective in reducing Pb and Cd concentrations in the bio-processed flour, when compared to As and Cr, which showed a higher stability rate. Interesting, the F11 specimens exhibited about a 50% reduction (cumulatively), in the four HMs analyzed in this research. The reduction in HMs concentration can be attributed to the remediation effects of the treatments. These treatments were able to facilitate the leaching of HMs from the cassava roots into the soaking water, as confirmed by the results presented in Table 2. Some of the metallic salts are fairly soluble in warm water, leading to the degradation of the HMs’ concentration (70). Moreover, bioactive compounds – including polyphenols, alkaloids, and organic acids, have strong chelating effects, which cause them to bind heavy metals (HMs) and produce stable complexes. This inhibits heavy metals absorption into the CF matrix, primarily by reducing their mobility and bioavailability potentials (71). Also, phytochemicals in the treatments enhance the enzymatic hydrolysis of HMs, which provides an effective decontamination effect; through increase precipitation and oxidation rates (36, 45, 60, 72).

**Table 6:** The heavy metals content of the CF specimens (μg/kg DW).

Sample code	Pb	Cd	As	Cr
Control A	68.67^i^ ± 3.21	63.00^g^ ± 2.65	11.13^h^ ± 0.80	68.00^g^ ± 3.00
Control B	61.33^h^ ± 2.08	57.67^f^ ± 1.53	9.97^g^ ± 0.15	65.00^f^ ± 1.00
F1	58.00^h^ ± 2.00	54.33^ef^ ± 1.53	9.43^g^ ± 0.06	64.33^ef^ ± 0.58
F2	53.33^g^ ± 1.53	52.67^e^ ± 1.15	8.73^f^ ± 0.15	62.67def ± 0.58
F3	52.00^fg^ ± 2.00	51.00^de^ ± 2.00	8.33^ef^ ± 0.25	62.00cde ± 1.00
F4	49.33^ef^ ± 1.53	45.67^c^ ± 1.53	7.93^de^ ± 0.15	60.00^bcd^ ± 1.00
F5	41.33^d^ ± 3.06	44.67^c^ ± 2.52	7.10^bc^ ± 0.20	57.33^ab^ ± 1.53
F6	33.33^c^ ± 1.53	39.33^b^ ± 1.53	6.57^ab^ ± 0.15	57.33^ab^ ± 1.15
F7	48.00^d^ ± 2.00	47.67^cd^ ± 2.08	8.60^f^ ± 0.26	61.67^cde^ ± 1.15
F8	34.67^c^ ± 1.53	44.00^c^ ± 2.00	7.37^cd^ ± 0.23	61.67^cde^ ± 1.53
F9	51.00^efg^ ± 2.00	45.67^c^ ± 4.16	7.17^bc^ ± 0.25	59.33^bc^ ± 2.08
F10	28.33^b^ ± 3.06	36.67^b^ ± 1.53	6.13^a^ ± 0.72	58.33^b^ ± 1.15
F11	21.33^a^ ± 1.53	27.33^a^ ± 2.52	6.07^a^ ± 0.15	55.33^a^ ± 1.53
WHO/FAO	300	200	200	NA

Mean ± standard deviation; *n* = 3; Rows with common letters (superscript) in the same column signified that there was no significant difference between the means (*p* ≤ 0.05).

The Pb, Cr, and Cd concentrations recorded across the various treatment groups, were less than the values reported by Dibofori-Orji and Edori (73) and Ande et al. (74), for cassava flour sampled from other geographical locations. Also, Anim et al. (25) reported a higher concentration of Pb in cassava sampled from remediated goldmine fields. However, the Pb concentrations achieved in this study, were similar to the results documented by Isaac et al. (75), for flour produced from *Dankasa* and *Shiga banza* cassava varieties. Furthermore, though the arsenic and cadmium values recorded in this research were generally lower than the findings of Ogah and Ozioma (76), their chromium concentrations were within the range of values documented in this study. In addition, Asomugha et al. (77) reported higher levels of As and Pb, although their Cd concentrations aligned with the results obtained in this research for the enriched CF samples. The inconsistency in HMs concentrations reported by various scholars, can be linked to geographical locations, field practices, type of treatment adopted, laboratory errors, and other anthropogenic factors (78).

These HMs (Pb, As, Cr, and Cd) are considered toxic metals, because they play insignificant roles in human nutrition and in the medical field. Rather, their trace concentrations in the body have many health complications. Lead poisoning can result in coma, neurological defects, anemia, and convulsions (25). Cadmium toxicity includes renal disorder and carcinogenicity (27); while arsenic toxicity has been linked to cardiovascular diseases and cancer (79). Additionally, higher chromium concentrations have been associated with respiratory and gastrointestinal problems (26). Interestingly, the results obtained in this study never exceeded the maximum permissible limits of 300 μg/kg DW for Pb, and 200 μg/kg DW for Cd and As, as approved by WHO/FAO (56, 80).

### Antioxidant properties

**Figure 2:**
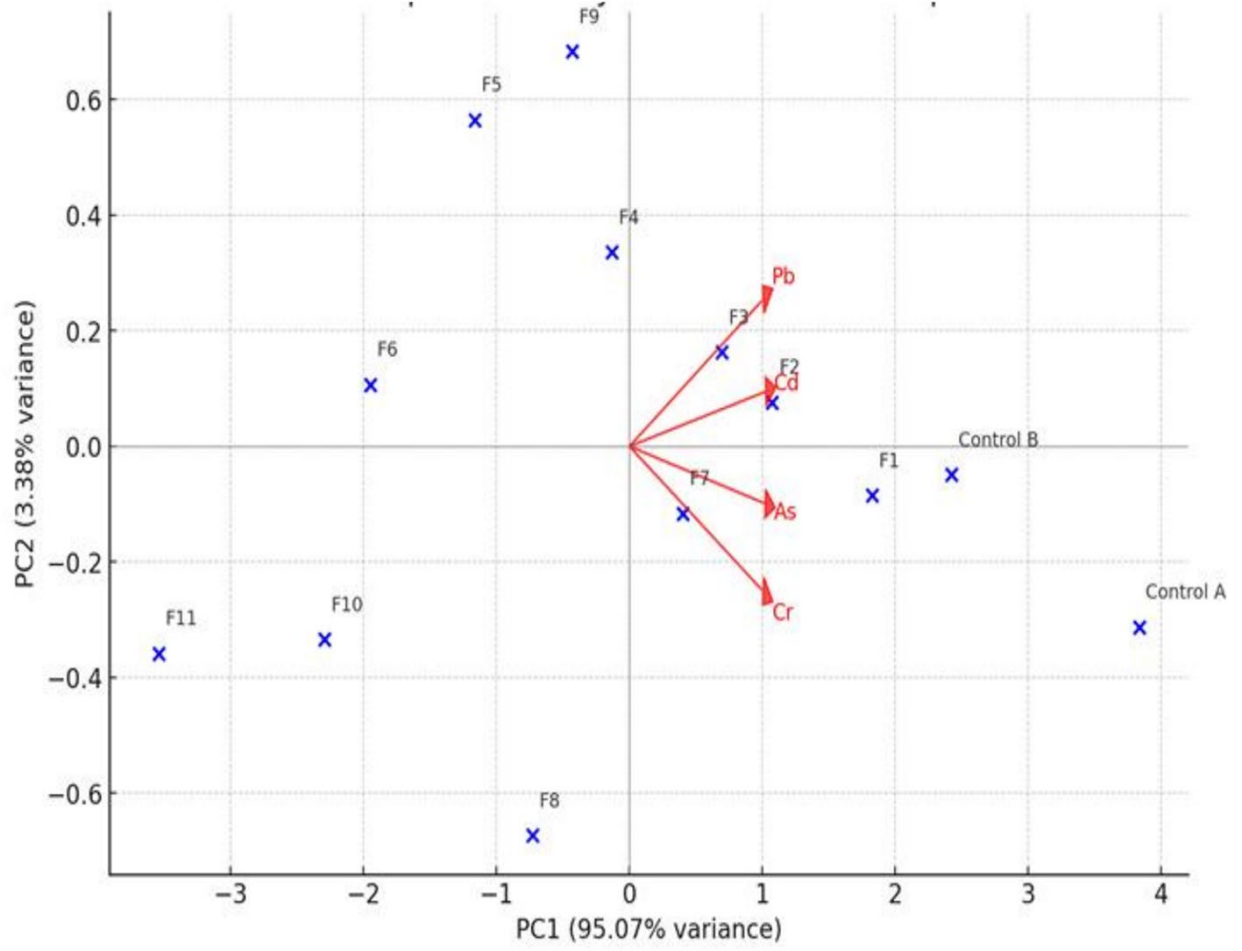
The PCA biplot of the heavy metals concentration and the CF samples. Pb, lead; Cd, cadmium; As, arsenic; Cr, chromium.

The total phenolic content (TPC), and DPPH radical scavenging activity (DRSA), of the cassava flour samples results are presented in Table 7 and Figures 2. It was noted that the applied treatments, had significant influence on the both parameters evaluated (*p* ≤ 0.05). It was noted that the carrier control unit, had the lowest TPC and DPPH values - 125.33 mg GAE/100 g DW and 34.33%, respectively, which can be linked to the leaching of the active phytochemicals into the plain soaking water. According to Plaskova and Mlcek (81), the soaking of cassava roots leads to their decreased antioxidant capacity, through the diffusion of essential phytochemicals from the plant material into the water. The TPC and DRSA results recorded in this study were greater, than those reported by Nilusha et al. (8), for CF samples produced from several cassava cultivars, which can be correlated with the treatments applied in this research. However, this study’s documented values similar the values stated by Bamidele (5) for microbial-assisted fermented CF, substantiating the antioxidant effects of the treatments.

**Table 7:** The antioxidant values of the CF samples.

Sample code	TPC (mg GAE/ 100 g DW)	DPPH (%)
Control A	170.67^e^ ± 5.51	49.00^de^ ± 3.61
Control B	125.33^a^ ± 2.52	34.33^a^ ± 3.21
F1	166.00^de^ ± 2.00	45.67^cd^ ± 2.08
F2	177.33^f^ ± 4.51	51.00^ef^ ± 2.00
F3	150.00^b^ ± 4.00	38.33^ab^ ± 1.53
F4	155.33^bc^ ± 4.51	44.33^c^ ± 2.52
F5	159.33^c^ ± 3.21	40.00^b^ ± 2.00
F6	171.00^e^ ± 3.00	49.33^de^ ± 1.53
F7	170.00^e^ ± 3.61	53.33^ef^ ± 2.08
F8	180.00^f^ ± 2.65	55.33^f^ ± 1.15
F9	160.67^cd^ ± 3.06	50.00^de^ ± 3.00
F10	191.33^g^ ± 3.21	66.00^g^ ± 3.61
F11	214.33^h^ ± 4.16	72.33^h^ ± 2.08

Mean ± standard deviation; *n* = 3; Rows with common letters (superscript) in the same column signified that there was no significant difference between the means (*p* ≤ 0.05).

Soaking with natural additives, alters the free radical scavenging activity of the cassava root flour, leading to the release of bioactive metabolites within the root, facilitating a biotransformation process and resulting in enhanced antioxidant activity (5, 82). Additionally, the experimental findings show that the treatments (F1–F11) demonstrated significant potential in enhancing both TPC and DPPH levels of the cassava flour. The results depicted that F10 and F11 samples exhibited the maximum TPC and DPPH values, signifying their enhanced antioxidant capacity and medicinal properties. Particularly, the higher amounts of antioxidants recorded in Treatments F7 to F11, can be linked to the larger concentrations of protein and vitamins documented in these particular treatments (Table 5).

Besides, there is a stronger tendency that the extracts will facilitate the bio-synthesis of some new phenolics through advanced hydrolysis of certain organic acids, which act as transitional compounds for secondary metabolic routes (83). This action can also be linked to the rapid increase observed in the TPC and antioxidant activity of the treated CF samples. Higher TPC and DPPH values help in preventing oxidative stress-related disorders (84). Muscolo et al. (85) stated that antioxidants play a major role in the prevention of chronic ailments such as cancer, diabetes, neurodegenerative, and cardiovascular disorders, by inhibiting the occurrence of oxidative stress and cells inflammation. Phenolic compounds encourage healthy gut performance, by enhancing appropriate microbial activity and sustaining microbial balance (5, 86).

According to Uguru et al. (69), the effects of organic additives are influenced by the phytochemical metabolisms of the bio-agents, which differ widely among the bio-extracts, leading to their unique antioxidant behaviors. These results highlighted that increasing the treatment’s concentration automatically leads to an increase in the antioxidant level, which has several nutritional and medicinal advantages. However, high concentrations of these extracts can have a negative impact on the flavor and textural profile of the flour, and the accumulation of toxic compounds and heavy metals in the flour, above the public health safe limits.

### Physiological index

The results of the glycemic index (GI) of the CF samples are presented in Table 8. It was noted that the soaking and bio-fortification have significant impacts on the CF samples GI values. The untreated sample (Control A) recorded the maximum GI value of 78.00, while the F11 specimen had the minimum GI value of 38.00. Particularly, the Control B specimen had a slightly lower GI value of 69.33, which suggests that soaking in plain water alone plays some critical role in reducing the GI value of the CF. Pronounced GI reduction (from 65.67 to 55.33) was recorded between samples from treatments F1 and F6, though the lowest GI values (50.33 to 38.00) were documented between samples from treatments F7 and F11. This highlighted that some of the treatments were more effective than others, and notably, the single extract-based treatments (F1 to F6), were not potent enough to drastically modify the CF glycemic behavior. Remarkably, the GI values reported in this study were similar to those obtained by Wylis Arief et al. (87) and Adefegha et al. (88) for bioprocessed cassava-based foods. However, Ogbuji and David-Chukwu (89) reported a higher GI value of 84.06 for fermented cassava roots, while Nwaliowe et al. (90) documented GI values that ranged from 86.42 to 89.09 for four cassava cultivars. Also, Eyinla et al. (91) reported a higher Glycemic Index (GI) value of 91 for the processed cassava product.

**Table 8:** The glycemic index values of the CF samples.

Sample code	GI level (unit less)
Control A	78.00^h^ ± 3.61
Control B	69.33^g^ ± 1.53
F1	63.67^ef^ ± 4.73
F2	55.33^d^ ± 2.08
F3	65.67^f^ ± 3.06
F4	60.67^e^ ± 2.81
F5	61.33^e^ ± 1.53
F6	57.67^d^ ± 2.08
F7	50.33^c^ ± 1.53
F8	47.67^bc^ ± 2.31
F9	53.33^cd^ ± 2.08
F10	44.67^b^ ± 2.52
F11	38.00^a^ ± 2.00

Mean ± standard deviation; *n* = 3; column having similar letter (superscript) signified that, these means did not exhibit significant difference (*p* ≤ 0.05).

The results revealed that the treatments were able to reduce the flour GI value to the safer limit range. It has been recommended that low GI foods have glycemic index values that range from 0 to 55, moderate GI foods range from 56 to 69, and high GI foods have values greater than or equal to 70 (92). Glycemic index is a nutritional hazard indicator, and its higher values tend to cause rapid and substantial spikes in blood sugar levels. Diets with lower GI values are highly appropriate, mostly for patients with diabetes and cardiovascular diseases (93). The reduction in the GI levels can be attributed to, the antioxidant properties of the treatments, leaching of soluble carbohydrates during soaking, and microbial actions on the cassava roots (94). Antioxidants obstruct *α*-amylase and α-glucosidase performances, which critical for glucose production. Additionally, antioxidants inhibit enzyme activity and starch hydrolysis, thereby reducing the glycemic index. Flavonoids, gingerols, phenolic acids, and curcuminoids have strong potential to impede glucose mobility in the bloodstream, by seriously interfering with the basic SGLT1 and GLUT2 actions (19, 95).

**Figure 3:**
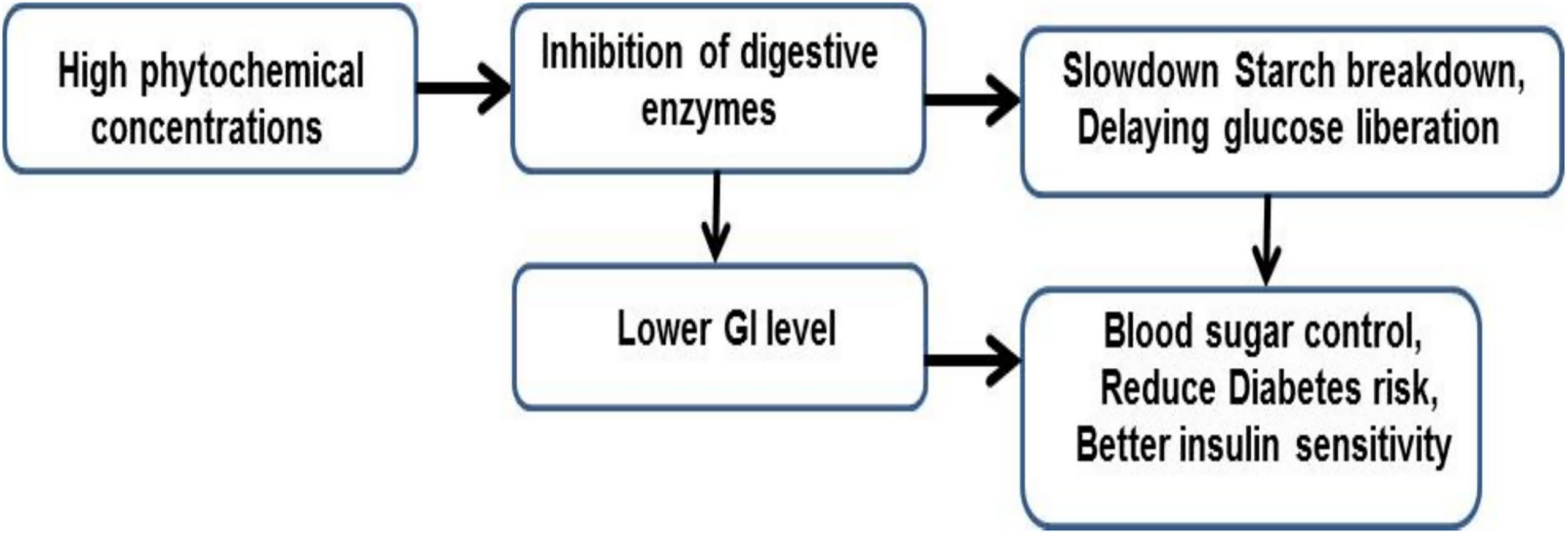
The conceptual flowchart of the GI degradation and health benefits.

Furthermore, Figure 3 shows the conceptual flowchart, illustrating how high phytochemical concentrations in the treatments suppress the glycemic index of the CF. It has been scientifically proven that flavonoids and phenolic acids retard α-amylase and α-glucosidase activity. This leads to a reduction in the rate at which the starch content of the flour is converted into glucose (96). Additionally, polyphenols are effective in improving insulin sensitivity and glucose utilization in the human body. Specifically, the reduction in glucose production will lead to lower glucose absorption into the bloodstream, resulting in the inhibition of rapid spikes in blood sugar. Consequently, this will provide greater health benefits against diabetes and obesity, making the enriched CF more appropriate for dietetic management of protracted diseases.

### Pearson correlation analysis of all the parameters

The results of the Pearson correlation analysis are presented in Table 9. The Pearson correlation revealed high positive correlations (r ≥ 0.90) between oxalates, tannins, carbohydrates, protein, vitamins, TPC, and DPPH levels of the enriched CF. On the contrary, there were negative correlations between the nutritional quality and antioxidant characteristics and the toxic metals. Additionally, there was a good relationship between the anti-nutrients, and the carbohydrate contents of the enriched CF. It can be seen that the anti-nutrients - phytates, oxalates, and tannins, have moderate to strong correlations among themselves, which is an indication that the treatments increase these anti-nutrients concentrations simultaneously. Elevated concentrations of oxalates, phytates, and tannins in diets tend to reduce essential minerals bioavailability, leading to nutrient deficiency diseases (97).

**Table 9:** The Pearson correlation results of the CF samples.

Response	Phytates	Oxalates	Tannins	Vitamin C	Carot	Vitamin B	Vitamin E	Carb	Protein	TPC	DPPH	HCN	GI	Pb	Cd	As	Cr	Fat
Phytates	1																	
Oxalates	0.77**	1																
Tannins	0.790**	0.886**	1															
Vitamin C	0.328*	0.658**	0.556**	1														
Carot	0.467**	0.637**	0.499**	0.797**	1													
Vitamin B	0.632**	0.894**	0.818**	0.837**	0.80**	1												
Vitamin E	0.314	0.736**	0.583**	0.840**	0.72**	0.871**	1											
Carb	0.872**	0.892**	0.907**	0.554**	0.54**	0.835**	0.58**	1										
Protein	0.573**	0.850**	0.796**	0.842**	0.82**	0.954**	0.89**	0.755**	1									
TPC	0.447**	0.748**	0.780**	0.838**	0.70**	0.898**	0.83**	0.708**	0.93**	1								
DPPH	0.305	0.714**	0.672**	0.673**	0.65**	0.810**	0.82**	0.544**	0.85**	0.81**	1							
HCN	0.408**	0.002	0.144	−0.653**	−0.47**	−0.306	−0.59**	0.130	−0.39*	−0.44**	−0.36*	1						
GI	0.136	−0.365*	−0.295	−0.777**	−0.52**	−0.593**	−0.827**	−0.214	−0.66**	−0.71**	−0.66**	0.836**	1					
Pb	0.003	−0.357*	−0.260	−0.850**	−0.79**	−0.634**	−0.73**	−0.235	−0.67**	−0.68**	−0.62**	0.840**	0.834**	1				
Cd	0.036	−0.335*	−0.206	−0.810**	−0.80**	−0.617**	−0.75**	−0.174	−0.69**	−0.67**	−0.60**	0.846**	0.822**	0.942**	1			
As	0.128	−0.214	−0.035	−0.766**	−0.72**	−0.489**	−0.68**	−0.073	−0.55**	−0.52**	−0.50**	0.895**	0.788**	0.910**	0.90**	1		
Cr	0.129	−0.143	0.037	−0.678**	−0.71**	−0.42**	−0.582**	−0.015	−0.049**	−0.46**	−0.39*	0.818**	0.666**	0.824**	0.90**	0.86**	1	
Fat	0.012	−0.461**	−0.438**	−0.729**	−0.52**	−0.66**	−0.791**	−0.319*	−0.72**	−0.75**	−0.71**	0.644**	0.858**	0.728**	0.78**	0.65**	0.53**	1

**Correlation is significant at the 0.01 level (2-tailed). *Correlation is significant at the 0.05 level (2-tailed). Carot, carotenoids; Carb, carbohydrates.

Also, the results revealed that there was a strong relationship among the vitamins (Carotenoids, Vitamins B, C, and E), which indicates that the treatments enhance the vitamins’ content of the CF. The vitamins were also observed to strongly correlate with the antioxidant parameters (TPC and DPPH) and protein content, affirming their synergistic role in enhancing the antioxidant activity of the fortified CF. Additionally, it was noted that the TPC and DPPH had an inverse relationship with HCN, but a moderate negative correlation with GI of the enriched CF samples; in contrast, the TPC and DPPH were found to have a very strong relationship with the CF protein content. This is an indication that the antioxidants performed dual functions: increasing the nutritional quality value of the CF, and also reducing the HCN toxicity and GI risks of the CF. Additionally, the Pearson correlation analysis established that there was a negative relationship (r – 0.4 to −0.8) between the toxic metals (Pb, Cd, As, and Cr) and the vitamins, proteins, and antioxidants (Table 9). This supports the idea that the bioactive extract treatments, help to inhibit the accumulation of toxic metals in the cassava flour. On the contrary, the heat map revealed that there was a strong relationship among the toxic metals, HCN, GI, and the fat content of the CF.

Notably, the protein has a very strong connection with the TPC. This demonstrates that extract treatments help improve the nutritional and antioxidant capacity of the CF, buttressing the previous findings of Moo-Huchin et al. (98). The heatmap emphasizes that there was a strong negative correlation between HCN and the vitamin content of the flour. Remarkably, the results highlighted that HCN and GI have negative relationship with the essential nutrients such as proteins and vitamins. This is one of the major goals of this research, which is reducing the cassava flour HCN and GI levels to safe limit, making it suitable for diabetic and other vulnerable patients. The results also depicted that the CF fat content has a moderate to strong negative relationship with the vitamins, antioxidants, and proteins. This depicts that the treatments help decrease the fat content of the CF and, at the same time, increase the antioxidant-nutrient balance of the flour, which is advantageous to human metabolic health.

This is affirmed by the strong positive correlations between the vitamins and protein, as shown in the Pearson correlation table. For instance, protein and B vitamins relationship (*r* = 0.954**), and protein and vitamin C relationship (*r* = 0.842**). Also, the TPC and DPPH were closely aligned, which indicates that these factors share a common antioxidant capacity and contribute to the health-promoting features of cassava flour. According to Kalogerakou and Antoniadou (99), antioxidants play essential roles in human health, including the enhancement of reproductive health, immune function, and healthy vision, together with neutralizing free radicals. On the contrary, the GI and fat content formed a cluster, whose position was almost opposite to that of the vitamins and antioxidants. Notably, this validates this study’s results, which reported that the flour’s GI and fat levels were inversely proportional to the concentrations of vitamins and antioxidants. This aligns with the Ansari et al. (100) report, which stated that large phytochemicals’ concentrations have the potential to lower the GI levels in diets, by inhibiting carbohydrate digestion rate, thereby lowering the blood glucose level.

### Principal component analysis

**Figure 4:**
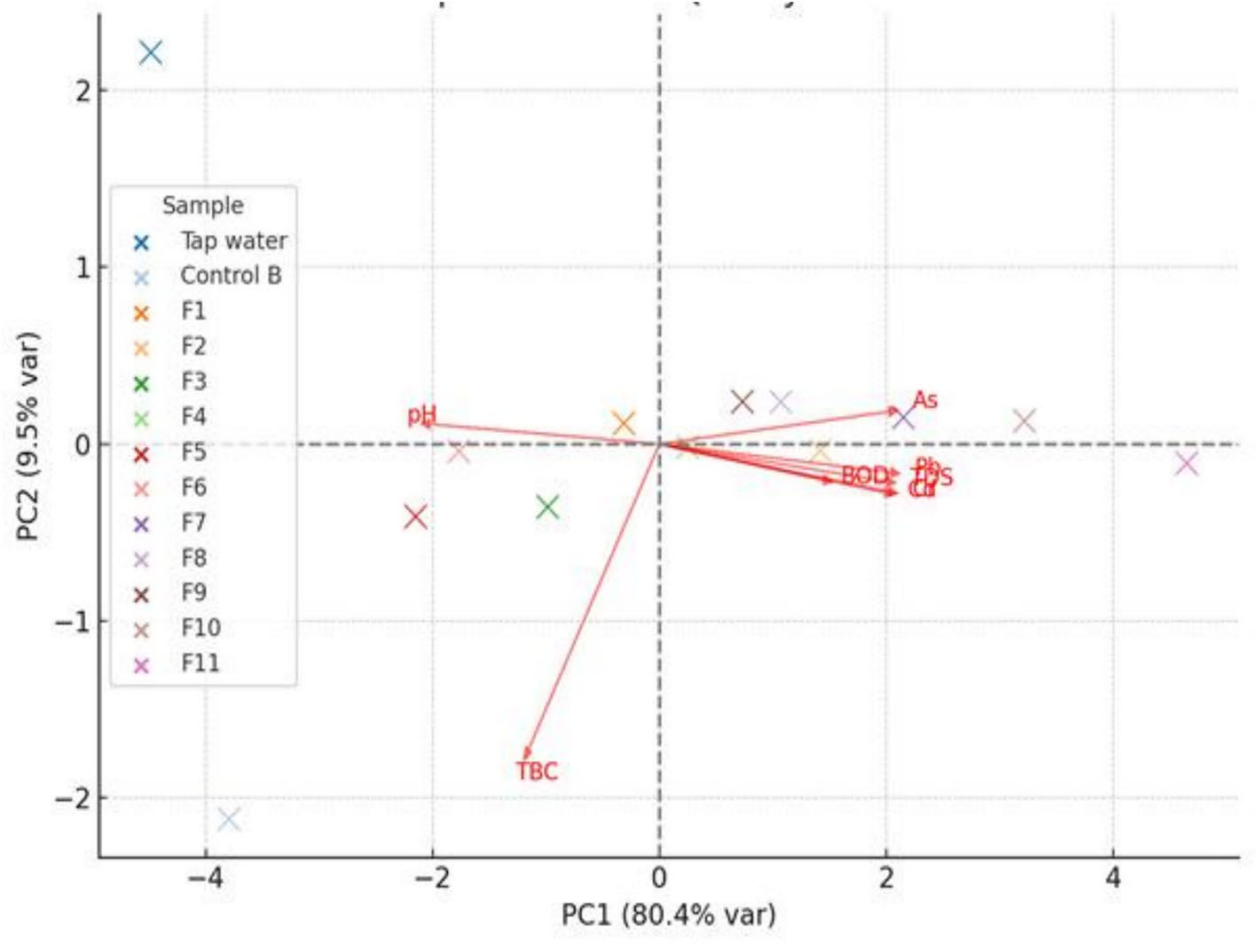
The PCA biplot of the cassava roots soaking water quality. Pb, lead; Cd, cadmium; As, arsenic; Cr, chromium; TDS, total dissolved solids; BOD, biochemical oxygen demand; F1 to F11, treatments 1 to 11; PC1, principal component 1; PC2, principal component 2.

**Figure 5:**
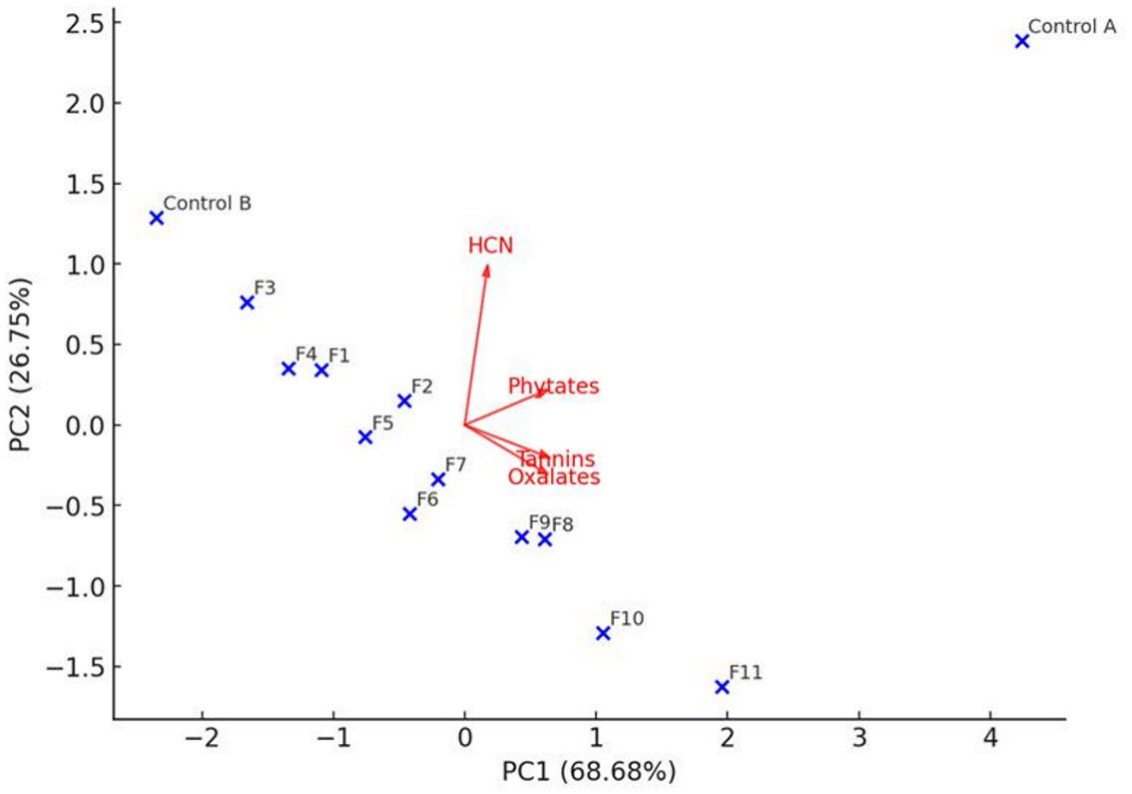
The PCA biplot of the anti-nutrients parameters of the cassava flour. Carb, carbohydrate.

**Figure 6:**
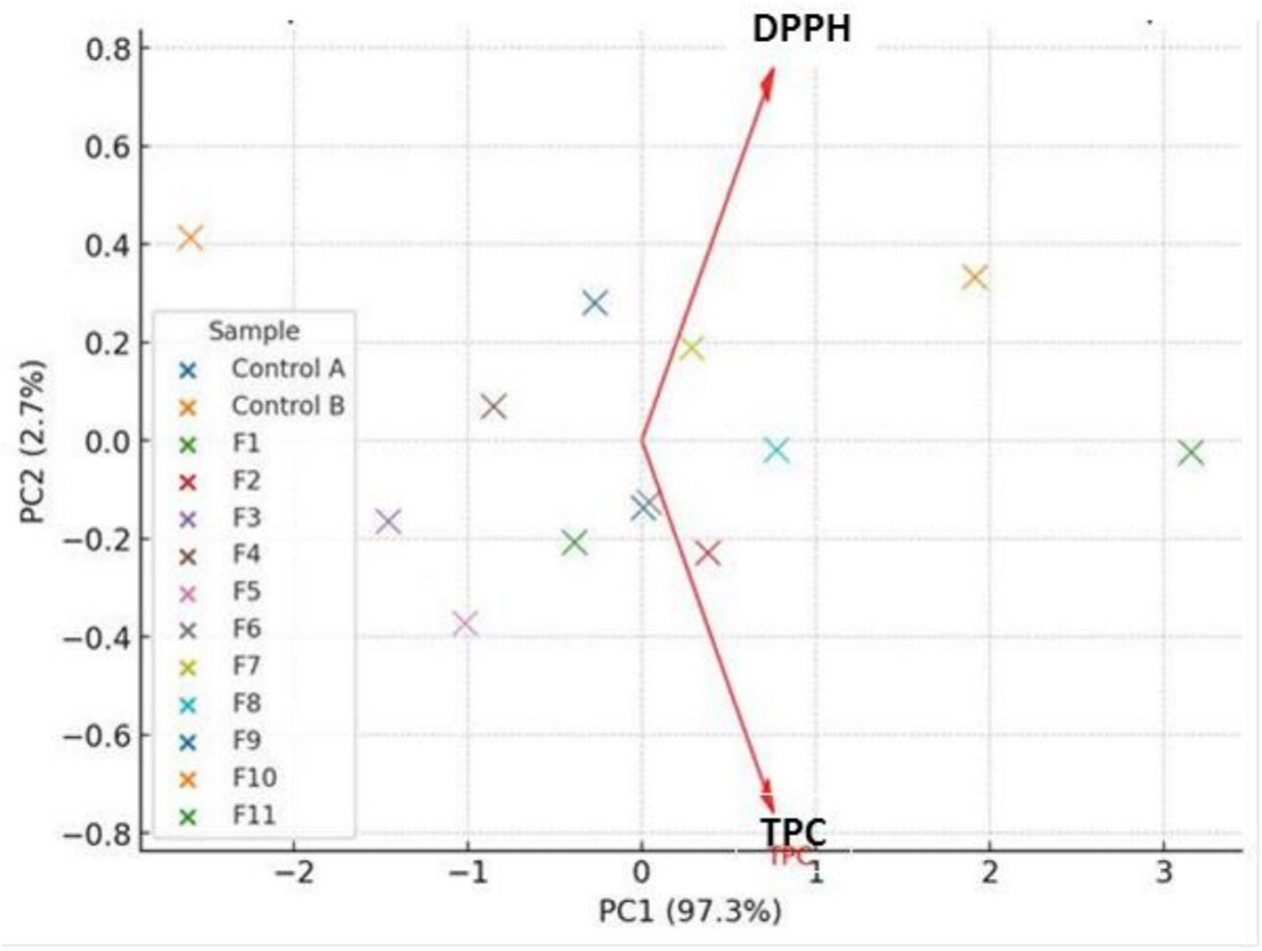
The antioxidants and cassava flour PCA biplot of the cassava flour. TPC, Total phenolic content.

The results of the PCA biplots are presented in Figures 4–6. Figure 4 affirmed that the treatment greatly affects the microbial performance and HMs content of the soaking water. For instance, the plain tap water was located apart from all the other points, which indicates that it has noticeably lower levels of HMs, TBC, TDS, and BOD, compared to the water samples from Control B and F1 through F11. Also, treatments F10 and F11 show a strong positive relationship with Cd, Pb, and Cr, as their cluster was strongly separated along the PC1 axis. Treatments F1, F2, and F7 show moderate HMs contamination, while treatments F5 and F6 were located around moderate pH and lower HMs contamination. On the contrary, the pH position was located opposite the BOD and TDS orientations, suggesting an inverse correlation - higher water acidity may lead to increase organic matter content, and greater heavy metals solubility. These observations corroborate Zhao et al. (48)‘s report on surface water quality. Conclusively, the PCA biplot has further affirmed that bio-extracts treatments had distinct influence on the water quality, resulting to elevated heavy metals accumulation, and inhibition of microbial activities. Since the water was not spiked with additional metallic or biological compounds, the variations in the concentrations of the parameter (primarily the increases in HMs and TDS), can be linked to the leaching of soluble compounds from the cassava roots into the soaking water.

The PCA biplot in Figure 5, further revealed the multivariate visualization of how the anti-nutritional parameters, varied among the treated and untreated cassava flour samples. It was observed that Control A is located at the extreme ends of both PC1 and PC2 axes, which confirms its extremely high levels of anti-nutrients. The stark separation of Control A from the other points further proved that, the soaking technique and other bio-additive treatments substantially altered the nutritional safety profile of the flour. Particularly, the PCA biplot obviously establishes that, the various treatments substantially modify the balance between HCN and other anti-nutrient compounds, which is essential for food security and public health. According to Chiwona-Karltun et al. (18) and Samtiya et al. (101), prolonging the soaking duration and complementary treatment, greatly helped reduce the HCN level of CF, as well as improving the flour’s nutritional value.

Figure 1 enhance better understanding of the effectiveness of the treatments. It was noted that protein, carotenoids, B vitamins, vitamin C, and vitamin E were located around the PC1 positive side, while fat was located on the PC1 negative side. This suggested that the essential nutrients were highly correlated, as well as their concentrations increased together in the same pattern. Additionally, the PCA results revealed that the carbohydrates behave slightly independently of the vitamins and protein behaviors. The Control A position indicated that this sample’s nutritional value, is significantly lower than that of samples F1 to F11, affirming its baseline composition. Also, treatments F1–F4 are clustered in the moderate nutrient region, which indicates that these enriched CF samples have moderate micronutrient concentrations. The F8–F11 enriched flour samples, particularly the F10 and F11 samples, are situated around the positive axes of PC1 and PC2, which indicates that there is a substantial enrichment of vitamins, proteins, and carotenoids in the flour, along with a lower fat content. The negative relationship between fat and other nutrients indicates that the treatments tend to enhance the dietary quality of the flour and reduce the less desirable nutrients.

Figure 2 further provides a better understanding of the interactions, between the CF specimens and the four toxic metals (Pb, Cd, As, Cr). For instance, the Pb and Cd locations suggested a strong positive correlation; hence, a reduction in one HM concentration, will lead to a decline in the second HM concentration. Notably, the PCA results showed that the HMs concentrations in treatments F1 to F4 remained closely related to that of the Control B. It was observed that the F11 sample was located far from the contamination vectors, indicating that this treatment plan, has the highest efficiency in degrading the toxic metals’ concentrations, especially Pb, Cd, and As.

Additionally, the Figure 6 shows that there was a direct proportional relationship, between TPC and DPPH, affirming the role of phenolic compounds as natural antioxidants. The increase in the antioxidants can be attributed to the improvement, of enzymatic reactions (hydrolysis and oxidation inhibition processes), which were facilitated by the presence of the plant extracts in the soaking water; thereby, promoting the breakdown of complex phytochemical compounds within the root tissues, into simpler bioavailable phenolic compounds (5).

### Limitations of the study

This study was based on only one cassava variety, harvested from one location in one planting season; hence the results obtained cannot be used to generalize cassava flour quality. This is because plant varieties, soil and climatic conditions, can significantly affect the biochemical properties of cassava roots. Though this research will not experience water quality variability as a limitation, since the tap water used was collected at once from the same tap, subsequent reproducibility of this research using tap water, may face water quality variability limitations. This is because ground water quality, such as: heavy metal content, physicochemical properties, and microbial composition - is highly dependent on factors like sampling location, depth, season, method, and storage conditions (102). Therefore, it is recommended that, future comprehensive research be carried out on multiple cassava cultivars, multiple planting seasons, and locations to obtain robust information and to develop broad-based, enriched cassava flour.

## Conclusion

This study was conducted to evaluate the importance of soaking, and bio-fortification on the nutritional quality, antioxidant capacity, anti-nutrient content, toxic metal levels, and physiological indices of cassava root flour (CF). Cassava roots were grouped into 13 experimental groups, of which 11 were subjected to different treatment options (F1 to F11), along with two controls (Control A and Control B). The biochemical properties of all the CF produced (both treated and untreated), as well as the soaking water, were tested in accordance with internationally approved standards. It was noted that the treatments has strong antioxidant and antimicrobial effects, on both the soaking water and the enriched CF. The findings showed that the organic extracts inhibited microbial activity, but increased the BOD and TDS levels of the cassava roots soaking water. Notably, the hybridized treatments substantially increased the fortified CF nutraceutical qualities, while reducing the toxic levels in the flour. It noted that the F10 and F11 treatments were the most effective treatments among all the treatment units. Particularly, in the F11 sample, the total phenolic content and carotenoids increased by 41.52 and 44.4%, respectively; while the HCN and glycemic index declined by 69.76 and 51.28%, respectively. Remarkably, the enriched CF samples are more valuable for health-conscious diets, especially for diabetic patients. On the contrary, the outcomes revealed that, the hybridized treatments caused a trade-off involving anti-nutritional factors, specifically phytates, oxalates, and tannins, in the enriched cassava flour samples. Basically, the research framework has established that a cost-effective bio-fortification technique, by utilizing locally sourced bio-additives and technology, can practically produce CF with enhanced nutritional values, lower toxicity, and higher public health safety. Therefore, resulting from the higher phytates, oxalates, and tannins levels in the enriched cassava flour documented in this study, more biofortification strategies should be further investigated upon. This is to minimize the accumulation of anti-nutrient concentrations in the treated CFs, while at the same time maintaining the environmental friendliness of the treatment approach. This will help produce bio-fortified CF, with wider and safer dietary and medical applications.

## Data Availability

The original contributions presented in the study are included in the article/supplementary material, further inquiries can be directed to the corresponding author/s.
